# From working collections to the World Germplasm Project: agricultural modernization and genetic conservation at the Rockefeller Foundation

**DOI:** 10.1007/s40656-017-0131-8

**Published:** 2017-03-20

**Authors:** Helen Anne Curry

**Affiliations:** Department of History and Philosophy of Science, University of Cambridge, Free School Lane, Cambridge, CB2 3RH UK

**Keywords:** Rockefeller Foundation, Seed banks, Crop diversity, Plant genetic resources, Green Revolution, Agricultural modernization

## Abstract

This paper charts the history of the Rockefeller Foundation’s participation in the collection and long-term preservation of genetic diversity in crop plants from the 1940s through the 1970s. In the decades following the launch of its agricultural program in Mexico in 1943, the Rockefeller Foundation figured prominently in the creation of world collections of key economic crops. Through the efforts of its administrators and staff, the foundation subsequently parlayed this experience into a leadership role in international efforts to conserve so-called plant genetic resources. Previous accounts of the Rockefeller Foundation’s interventions in international agricultural development have focused on the outcomes prioritized by foundation staff and administrators as they launched assistance programs and especially their characterization of the peoples and “problems” they encountered abroad. This paper highlights instead how foundation administrators and staff responded to a newly emergent international agricultural concern—the loss of crop genetic diversity. Charting the foundation’s responses to this concern, which developed only after agricultural modernization had begun and was understood to be produced by the successes of the foundation’s own agricultural assistance programs, allows for greater interrogation of how the foundation understood and projected its central position in international agricultural research activities by the 1970s.

## Introduction

In 1943, the Rockefeller Foundation launched a program in Mexico that aimed to increase agricultural production through greater research and training in the agricultural sciences. Many scholars have looked to this effort as the starting point of the so-called Green Revolution—that is, of the striking increase seen in the production of certain economic crops in Latin America and Asia in the late 1950s and 1960s that resulted especially from the introduction of higher-yielding crop varieties and the agricultural technologies and financial structures needed to cultivate these (Jennings 1988; Perkins 1997; Cotter 2003; Harwood 2009, 2012; Cullather 2010; Patel 2013). In establishing this narrative, historians have traced the foundation’s efforts from Mexico across Latin America in the 1940s and 50s, to India, Pakistan and elsewhere in Asia in the 1960s and 70s, and finally to Africa where these faltered in the 1980s and later (Oasa 1981; Fitzgerald 1986; Matchett 2002, 2006; Cullather 2004; Shepherd 2005; Smith 2009; Baranski 2015). The outcomes of these activities remain contested, hailed by some as having prevented global food crises and decried by others as the source of innumerable social and environmental problems.^1^

Much of the historical literature that has evaluated the agricultural assistance programs of the Rockefeller Foundation from the 1940s onward has focused on the outcomes that were vigorously pursued from the outset. These include especially the creation and dissemination of high-yielding varieties of key economic crops and the broad political agendas, such as transforming potentially volatile peasants into cooperative capitalist farmers, that were encoded in seemingly narrow breeding efforts. Some of these studies have explored how the prior experiences and assumptions of foundation officers and staff affected what they defined as problems worthy of attention in the countries they visited as well as the solutions they proposed (e.g., Fitzgerald 1986; Shepherd 2005; Smith 2009; Nally and Taylor 2015). The perspectives of foundation officers about the need for agricultural assistance and the form it should take were not developed in isolation but reflected contemporary theories of economic development and were bound closely to US geopolitical concerns and strategies for national security in the midst of the Cold War; these, too, have been consistent themes in studies of the Rockefeller Foundation’s agricultural programs (e.g., Oasa 1981; Perkins 1997; Cullather 2010).

Although it complements these many studies, the history that I relate here differs in its approach to understanding the post-1940 agricultural programming of the Rockefeller Foundation and its wide-ranging effects. I explore a component of the foundation’s interventions in global agriculture—its participation in the collection and long-term preservation of genetic diversity in agricultural plants—that was not prompted by the perceived shortcomings of developing-world agriculturists or governments but instead arose as a means of managing some less desirable consequences of the foundation’s own activities. As I describe here, in the decades following the launch of the agricultural program in Mexico in 1943, the Rockefeller Foundation figured prominently in the creation of collections in which many thousands of varieties of important economic crops were gathered. I show how, through the efforts of its administrators and staff, the foundation subsequently parlayed this experience into a leadership role in international efforts to conserve “plant genetic resources” as these coalesced in the late 1960s.

These activities have had long-term—indeed, continuing—consequences for international agricultural research and development. The institutions favored by the Rockefeller Foundation as centers of collection and conservation, whose histories I recount here, still play central roles in the management and global circulation of plant genetic materials, especially for key economic crops such as rice, maize and wheat.^2^

The history of the Rockefeller Foundation’s involvement in the collection and long-term maintenance of crop genetic diversity aligns with the existing scholarship in showing the influence of the foundation’s resources and vision on crucial elements of agricultural production at local and global scales. Yet because of the particular nature of the problem of the loss of genetically diverse types, exploring this history allows for greater interrogation of how the foundation (as embodied in its staff) came to understand and position itself as an undisputed authority in international agricultural research. This is because the loss of genetically diverse types became an urgent international concern only *after* agricultural modernization had begun, and was in fact understood to be produced by the very successes of the foundation’s agricultural programs. Here the problem to be solved could not be explained by reference to the failures of backwards farming communities or addressed by imposing an imported vision of good agriculture. It was acknowledged to be a product of the foundation’s own activities. What’s more, it invited the interest of other organizations, who found themselves engaged in resolving an issue seen to be exacerbated by agricultural modernization as pursued by the Rockefeller Foundation. As a result, this history offers an opportunity to consider how the foundation viewed its authority in the domain of global agriculture vis-à-vis that of other national and international actors.

## Mexican maize diversity

The Rockefeller Foundation’s first forays into the collection of crop plant diversity in the 1940s were intended not as conservation activities but rather as the initial steps towards specific agricultural objectives: the creation and distribution of higher-yielding crop plants for Mexican farmers. Within a few short years, however, concerns about the potential loss of the diverse types of maize (corn) found in Mexico and elsewhere in Latin America transformed a working collection—that is, one used for the immediate research needs of breeders and other scientists on site—into a resource to be expanded and stewarded for the future.

In October 1943 the Rockefeller Foundation entered into an agreement with the Mexican government in which it agreed to provide Mexico with technical expertise in agricultural research, with the aim of improving national agricultural productivity, via a new American-staffed Office of Special Studies in the Ministry of Agriculture. It followed that a top priority for this office was to establish programs in plant breeding, as the development of high-yielding varieties of important economic and subsistence crops like maize, wheat, and beans promised a straightforward route to increased agricultural production.^3^

One of the first activities to be undertaken in the maize-breeding program, which was directed by the American geneticist and breeder Edwin Wellhausen, was to establish a collection of locally adapted Mexican maize varieties (today called landraces). The stated purpose of collecting was to support the maize improvement efforts; however, it was further encouraged by more academic interests. As a consultant to the Rockefeller Foundation, the Harvard botanist and maize breeder Paul Mangelsdorf had been instrumental first in shaping the Mexican program, then in launching the Office of Special Studies and setting an agenda for its maize program (Matchett 2002). He had also been engaged in the study of maize’s evolutionary origins for a number of years, work in which he relied on varieties gathered from across the Americas. His passion for both breeding and evolutionary studies influenced subsequent ambitions for and uses of the collection (Mangelsdorf 1974).

Foundation staff made the first collections of maize while driving across the Mexican countryside. According to Wellhausen, they gathered many varieties, “right along the road, as we went from one place to another.”^4^ Over time, the collecting activities became more methodical. Wellhausen was soon dispatching students from the nearby university to gather maize from all corners of the country. They were provided with “bags and tags and money to travel” and relied on buses and sometimes burros to travel to what Wellhausen called “the hinterlands.”^5^ Once they had arrived at remote sites, collectors often faced a still more difficult task. Collecting diverse types of maize frequently meant negotiating with impoverished farmers for access to a precious resource.^6^

The collectors’ persistence paid off. In 1943, when the collections had only just begun, Wellhausen reported the “very large number” of more than 200 maize types held by the Office of Special Studies.^7^ By 1947 the collection included some “1500 varieties of corn” and Wellhausen and his colleagues, encouraged by Mangelsdorf, had begun an effort to classify these and definitively establish their relationships to one another.^8^

Almost from the outset those associated with the Office of Special Studies recognized that their maize-breeding program, if it were successful, would produce more desirable strains of maize that might well replace the many diverse types they now encountered in Mexican fields. This loss would be a blow to breeding efforts and botanical studies alike. When Wellhausen and his colleagues published the results of their classificatory effort, they both acknowledged this concern and assigned themselves a central role in addressing it: “The modern corn breeder… has a responsibility not only to improve the maize in the country in which he works, but also to recognize, to describe, and to preserve for future use, the varieties and races which his own improved productions tend to replace and in some cases to extinguish” (Wellhausen et al. 1952, foreword).

As it turned out, this notion was more widely shared. In 1949 the German geneticist Friedrich Brieger of the Universidade de São Paulo voiced his concern over the likely disappearance of maize varieties across the Americas to the botanist Ralph Cleland, who was then the chairman of the Division of Biology and Agriculture of the US National Research Council (NRC).^9^ Brieger’s dire predictions of varieties being lost within ten years inspired Cleland to assess interest in a conservation effort among his colleagues and potential funders. Within a couple of years, a small group of US scientists had formed a “Committee on the Preservation of Indigenous Strains of Maize” (hereafter, Maize Committee) under the auspices of the NRC (Clark 1956; Curry 2017). Its initial members included a number of botanists and geneticists, including Mangelsdorf, whose research had already sparked their interest in maize diversity. Convinced of the likely loss of diverse—and valuable—maize varieties resulting from the introduction of improved lines, members of the Maize Committee obtained a grant from the US Technical Cooperation Administration (TCA) to finance a hemispheric effort to collect and preserve these imperiled varieties. Their arguments for why an agency concerned with offering “technical assistance” to developing countries should support their work emphasized the essential role that the endangered varieties would play in future maize-improvement efforts across Latin America; however, they also called attention to the benefits to US agriculture and to scientific research.^10^

The Maize Committee, relying especially on Mangelsdorf as an intermediary, also sought to secure the aid of the Rockefeller Foundation on the ground in Latin America. J. George Harrar, the director of the program in Mexico, and his colleagues clearly saw the value of the Maize Committee’s work for their future activities. As Harrar argued to the foundation president Chester Bernard, “The importance of maintaining viable germplasm of native crop plants such as corn is obvious and of tremendous significance in the development of new varieties of corn to meet future problems.”^11^ He pointed out that the foundation had been doing this work for years, spending considerable sums in the creation of a maize collection in Mexico and, more recently, in Colombia where a second Latin American program had been launched in 1950. They were, in his assessment, in possession of unparalleled resources: “If we accept the proposal of the NRC committee we will immediately make available to them the most important maize material now in existence.”^12^ Bernard agreed with Harrar, but expressed reservations that the committee had not adequately considered the question of maintaining these varieties in the long-term. He worried that this expensive commitment would devolve to the foundation and insisted that the foundation not commit itself to providing for the indefinite maintenance of the collected maize.^13^

On that understanding, the Rockefeller Foundation signed on to the Maize Committee’s planned program. Over the next few years, the foundation supervised collecting missions and provided land and space for multiplying and renewing collected seeds. The NRC, using the $85,000 grant from the TCA, paid for equipment, supplies, travel expenses, and salaries associated with collecting. TCA funds also supported the creation of three storage centers to house the collections—located in Mexico, Colombia, and Brazil—with additional “stand-by” storage at a US Department of Agriculture (USDA) site in Maryland. Both the Mexican and Colombian centers were linked to Rockefeller Foundation operations. The Mexican storage facility was at Chapingo, the field site of the Office of Special Studies and home to Wellhausen’s earlier maize collections, while the Colombian storage facility was sited at the new Rockefeller Foundation outpost in Medellín.^14^

The Maize Committee quickly achieved its goal of amassing seeds of diverse types. By 1955, the committee claimed a total of 11,353 samples of maize from across the Americas, 10,922 of which had been officially catalogued and many thousands of which were duplicated in stand-by storage.^15^

This success created new problems, as the question of long-term maintenance and renewal of these collections in Latin America lingered. Although the Maize Committee had hoped to establish an endowment to support the storage centers in perpetuity, no donor materialized. In a heated exchange of 1953, foundation staff including Harrar took great offense at the Maize Committee’s (to their minds) cavalier assumption, outlined in a further grant proposal, that the foundation would go on maintaining and distributing the collected maize varieties at its own expense.^16^ Yet this is what eventually happened.

## New crops, new collections, new concerns

During the 1960s Rockefeller Foundation staff continued to emphasize the importance of collections of crop diversity to the success of crop improvement programs, and increasingly saw a clear role for the foundation in maintaining these collections. This was due in part to the foundation’s sponsorship of a greater number of agricultural initiatives. As it expanded its horizons from Mexico and Colombia to all of Latin America and then to South and Southeast Asia and beyond, the foundation generated a need for access to more and different crops. Perhaps more important, as staff members from the flagship Mexico program fanned out to these new sites, they brought with them a sense of the important role that the collection of diverse varieties had played in the successes of the Mexican program—and they endeavored to repeat that experience.

In 1956, in the midst of the maize collecting efforts in Latin America, the Rockefeller Foundation entered into an agreement with the Government of India to create a cooperative research program at the Indian Agricultural Research Institute in New Delhi. As the program got off the ground in 1957 and 1958, a maize-breeding initiative was the first to be established, followed shortly by an effort to improve sorghum and millets (Perkins 1997, pp. 153–154). Kenneth Rachie, formerly of the Office of Special Studies in Mexico, led the latter project. He immediately proposed collecting sorghum varieties just as “extensive collections of corn germ plasm” had been made in Latin America. “It is quite obvious that such collections played an important part in excellent development of maize improvement programs in Mexico and Colombia,” he noted. Although Rachie envisioned that Indian materials would be the starting point for the collections, he hoped that “introductions from other countries would be added … and eventually a world sorghum bank would be established.”^17^ Rachie considered this to be in the known remit of the foundation: “Establishment of world germplasm banks has been an important function of the Rockefeller Foundation agricultural programs in the past.”^18^ Rachie soon organized an India-wide collecting program in which Indian collectors sought out locally adapted varieties of sorghum, maize, and millets. He further supplemented the collection with materials from other institutions, as well as a sorghum collection that he had brought with him from Mexico, to create his envisioned “world collection” (House 1980, p. 98).

A similar tale unfolded beginning in 1960 at a different Rockefeller-linked institution, the International Rice Research Institute (IRRI) in the Philippines. Jointly funded by the Rockefeller and Ford Foundations, and directed initially by a former staff member of the Office of Special Studies, IRRI’s mission was to raise rice production across Asia through enhanced scientific research and training. As with the program in Mexico, breeding high-yielding varieties took center stage. And, as with the earlier maize and sorghum efforts, rice breeding began with an effort to assemble at IRRI as many rice varieties as possible. Because many agricultural institutions already possessed large collections of rice varieties, the primary method of collecting was to write to these institutions to see whether they would share their materials. By the end of 1972, the collection included nearly 7000 accessions, and had benefited from the donation of extensive materials from the USDA, the UN Food and Agriculture Organization (FAO), Japan, and Taiwan (Chandler 1992, pp. 103–104).

The brief overviews of the collecting programs offered here—especially of maize in the Americas and sorghum, maize, and millets in India—give little sense of the labor and resources required to assemble and maintain a collection of diverse crop varieties, even for a circumscribed region. To gather the sorghum, maize, and millet varieties of India, Rachie relied on seven lead field collectors from India. Prior to visiting any area Rachie and his collaborators had to gather information about the nature and location of crops likely to be found there, as well as the predicted time of harvest; they also had to contact local agricultural officials and receive permission, and in many cases assistance, to carry out the work of collecting. Taking a sample involved gathering the seeds, wrapping these in cloth and bagging them, completing a standardized data form in duplicate, packing the bags into further metal containers or burlap bags, and sending these back to New Delhi. As Rachie summarized, “Field collecting was found to be hard, exhausting work.” The collectors nonetheless accumulated 2463 samples of sorghum, 1582 of maize, and 2165 of varied millets—having spent a collective 674 days in the field and an additional 116 days planning the missions and having traveled an estimated 79,118 miles while in the field.^19^

Collecting was indeed hard, exhausting work. But, in many respects, maintenance proved harder still. It was only after seeds arrived at a storage facility that the never-ending tasks of maintenance began—processing seeds for storage, classifying the accessions, developing a system for tracking these and storing data, growing out seeds to regenerate stocks, making samples available to breeders worldwide and so on. An illustration of just how difficult maintenance proved is the history of the Latin American maize collection in the mid 1960s. This collection had been shepherded by the Rockefeller Foundation from the days of the Maize Committee through the closing of the Office of Special Studies in 1959 and then the inauguration of a new Inter-American Maize Improvement Program the same year. As institutional histories proudly note, the collection held by this latter program became the core material for the “genebank” of a new International Center for the Improvement of Wheat and Maize (CIMMYT) established in 1966 under an agreement between the Rockefeller Foundation and the Mexican Government (Taba et al. 2004). But what was the collection like at that time? As Mario Gutiérrez, the scientist hired to curate the CIMMYT genebank, later described, “What CIMMYT had in 1967 could not be classified as a bank but merely as a dump.” Seeds produced for the bank sat idly in tin cans awaiting proper storage, the refrigeration equipment was broken, and within the cold rooms disorganization reigned. According to Gutiérrez, “No true inventory existed and staff members had to spend sometimes as much as a week trying to locate the seed they wished.”^20^ Collecting, therefore, was only the beginning—and in some sense, the comparatively easy beginning—of a much more arduous task.

Though the collections may not have been as perfect as their originators would have wished them to be, and the task of maintenance an ongoing challenge, in the 1960s the international agricultural research institutes (CIMMYT and IRRI) and the agricultural program in India had some of the most comprehensive collections of varietal diversity in key economic crops found anywhere in world.^21^ The Rockefeller Foundation, as patron, had played a central role in bringing these into being.

## An international gene bank and the World Germplasm Project

While programs supported by the Rockefeller Foundation were accumulating massive numbers of varieties of select crops for the primary (though not exclusive) use of agricultural researchers affiliated with these programs, a growing number of breeders, botanists, and other biologists had begun to make calls for more, and more extensive, such collections. The 1960s saw increasing agitation for international efforts to gather and protect in perpetuity the vast genetic diversity in agricultural plants—and not just a few key crops but a wide range of species important to human social and economic activities. These calls prompted Rockefeller Foundation staff to develop, for the first time, an overarching vision for the foundation’s role in stewarding collections of crop varieties and to articulate a position on this newly international and now more strongly conservation-oriented issue.

What was behind this heightened concern for crop diversity? One short answer is the work of the international agricultural research institutes and other development initiatives. By encouraging farmers to transition to high-yielding varieties as part of a larger package of agricultural change, programs such as the those of the Rockefeller Foundation in Mexico and India and institutions like CIMMYT and IRRI were seen as having expedited a transition away from the use of genetically diverse landraces that agriculturists had noted and worried about since the late-nineteenth century. For some observers, these worries were further stimulated by a more general concern about ecological change; as a Dutch agricultural official described in 1973, “This is not only a matter of agriculture and developing countries but also a matter of… the concern of so many people today with our environment.”^22^

Of those organizations that responded to concern about the loss of genetic diversity in crop plants, two of the most important were FAO and an international research initiative called the International Biological Programme (IBP). It was members of an IBP committee who first produced a categorization of threatened “genetic resources” and indicated priorities for conservation within these. Hoping to transform their assessment into action, at least in the realm of agriculture, IBP members reached out to FAO (Frankel and Bennett 1970a, preface; Pistorius 1997, ch. 1–2). Founded in October 1945 as a permanent UN agency in the domain of food and agriculture, FAO had played a role in the coordination of plant exploration and the creation of global catalogues of varieties of major food crops since the late 1940s. The joint efforts of the IBP and FAO led to a 1967 FAO/IBP Technical Conference on the Exploration, Utilization and Conservation of Plant Genetic Resources, the aims of which were to chart conservation concerns related to “plant genetic resources,” survey current activities, and create a plan of action for the future (Frankel and Bennett 1970a; Pistorius 1997, ch. 2).

The plan elaborated at the 1967 conference was focused on stemming the perceived tide of losses. The first step would be to produce a survey of “genetic resources in the field”—that is, an assessment of the location and extent of “primitive material” (landraces) and the wild relatives of crops (also considered useful as potential breeding material). This global survey, coordinated through a central agency, would in turn serve as a guide for collecting the plants identified. Another survey would chart existing collections at institutions throughout the world. All of these genetic resources would then be classified, evaluated, documented, and conserved for the long term. The key recommendation of the 1967 conference was the creation or designation of “international seed storage facilities,” in particular an “international gene bank… available to all nations.” Finally, bringing all of these linked elements together would necessitate high-level coordination and oversight. Having agreed that this “can only come from [a] United Nations agency,” the summary indicated that FAO and its Unit on Crop Ecology and Genetic Resources and Genetic Resources Information Centre would be the obvious choice (Frankel and Bennett 1970b, pp. 13–17).

Although FAO may have seemed the logical institution to nominate as coordinating body for these activities, its status as a UN agency and mandate to improve food and agricultural production worldwide did not translate into clear international authority and effectiveness. The early ambitions for FAO to address world hunger through direct measures such as the reorganization of commodity markets, emergency food relief, and credit schemes to foster agricultural development were forestalled especially by British and US policymakers. US officials in particular wanted to promote free markets and, in the midst of the Cold War, retain the use of food aid as means of influencing leaders of developing nations and fostering goodwill abroad. As a result, FAO never received the funding or powers it needed to tackle hunger head-on, and its role in its early decades was limited largely to offering technical assistance and compiling statistics (Marchisio and Di Blase 1991; Staples 2006).

In the late 1960s, limited financial resources and authority remained a constraint on FAO activities. Although it had increased its operational programming, its technical assistance remained, according to one historical assessment, “modest in comparison to other, particularly bilateral, sources” of such assistance (Marchisio and Di Blase 1991, p. 61). Perhaps equally troublesome for the proposals of the 1967 conference, these years represented a period of transition at FAO. A multifaceted campaign against hunger begun in the early 1960s was curtailed in favor of a narrower focus on scientific and technical assistance and, under new leadership, FAO restructured both its internal organization and its programming priorities (Marchisio and Di Blase 1991, pp. 72–73; Staples 2006, p. 121). In the midst of these changes, the ambitious conservation agenda articulated at the FAO/IBP Technical Conference and envisioned as a task for FAO remained on paper, even as interest in the issue of plant genetic resources continued to mount in both national and international circles.

Confronted by this growing international concern, which not only emerged from the Rockefeller Foundation’s past activities but also potentially threatened its future work, foundation administrators moved to position themselves and their agricultural institutes as the most knowledgeable and effective agents in managing global crop diversity. However, they characterized this issue not as one of general conservation action, intended foremost to prevent a cascade of losses in crop species and their wild relatives, but rather as a targeted need to locate and make available to breeders increasingly rare “indigenous varieties” of major crops.

The director of the foundation’s agricultural sciences program, Sterling Wortman, offered a first formal assessment in March 1969. “During the past 25 years, The Rockefeller Foundation has contributed to science and to world agriculture by assisting in the collection, storage and evaluation of the world’s basic food crops—corn, wheat, rice, sorghum, and the millets,” Wortman noted. Yet further work remained to be done to “complete” collection and evaluation in these crops “before the indigenous varieties are further displaced by newer high-yielding types.” Wortman suggested that it would take two years for a group of experts to generate a status report on existing collections worldwide, which would then serve as a guide for gathering what diversity remained. He further indicated that the established international agricultural institutes (e.g., CIMMYT and IRRI) would be central hubs for these activities, organizing the collections and ensuring their availability to “the scientists who can make best use of them.”^23^

Why was such an effort required of the Rockefeller Foundation? Subsequent summaries emphasized that the foundation’s successes had drawn on collections of genetic diversity and that future efforts would depend on similar resources. “Results attained through the use of… genetically diverse resources have been spectacular, particularly in rice, wheat, and maize,” summarized one proposal, referring to the foundation’s programs in Latin America and Asia. “To maintain the achieved high levels of production of these and for the improvement of other basic food crops, we are entirely dependent on the availability of material in the germplasm banks.”^24^

Another important reason for the foundation to develop an overarching plan for the management of crop diversity was a widespread perception that they had played a role in placing such resources at risk. Even if it did not come up explicitly in many foundation reports and proposals, it always lurked between the lines. How could it not, when one of the most common examples offered to indicate the urgency of creating new collections was the spread of the Rockefeller-sponsored wheat and rice varieties?^25^ Foundation officers were well aware of this view.^26^ As such, the foundation was not just addressing an unfolding agricultural problem that it had the experience and resources to take on. It was addressing a problem generated in part through its own activities.

Finally, foundation officers may well have worried about other institutions encroaching on activities (the collection and maintenance of landraces) that were not only areas of extensive investment on the part of the foundation, but also tasks central to the identity and importance of the agricultural research institutes. Although Wortman and his colleagues recognized that cooperation with institutions such as FAO would be essential in any international germplasm conservation effort, they tellingly did not reach out to these institutions in their initial planning. Instead, they dismissed them. The consensus of an April 1969 meeting, for example, was that the FAO/IBP proposals had arrived at a dead end—they would “remain on paper.”^27^

Empowered by their experiences in this field, concerned about the future viability of their programs, cognizant of their role in imperiling genetic resources, and likely aiming to continue as leaders in a newly crowded enterprise, those in the Rockefeller Foundation’s agricultural sciences program forged ahead. In short order, they nominated four expert individuals—one each for maize, rice, wheat, and sorghum and millets—who in turn assembled a group that he and Foundation staff considered the best qualified to collectively establish the status of existing collections, gaps in these collections, and plans needed to ensure “continued and improved protection” of global germplasm resources.^28^

The summary statements to emerge from these efforts converged on a few themes. The first was that many varieties were indeed in danger of disappearing and further collection activities should be undertaken. A second was that oversight of collecting and maintenance should be done on a crop-specific basis and in most cases left to the care of the established agricultural research institutes. The wheat committee, for example, argued that CIMMYT, because of its “unique character and role as an international center of research… would be the logical institution to serve as the coordinator” for the activities recommended by the committee. Rather than attempt to build a centralized collection, or even a centralized effort, the committees generally agreed that each institute was best left to undertake independently the conservation efforts that its researchers felt most desirable.^29^

In April 1972, the Rockefeller Foundation dedicated the comparatively modest sum of $350,000 “toward the costs of completing the collection of the world germplasm of corn, wheat, rice, sorghum, and millets,” stipulating that this was to be used within a three-year period. The assumed recipients of this grant money were IRRI (for work on rice), CIMMYT (for wheat and maize), an as-yet-only-proposed International Center for Research in the Semi-Arid Tropics (later ICRISAT; for sorghum and the millets), and the Plant Introduction Research office of the USDA (presumably for collecting missions).^30^

The limited plan for protecting “world germplasm” formulated at the Rockefeller Foundation by 1972 was a far cry from the ambitious vision articulated at the FAO/IBP Technical Conference a few years earlier. It aimed at creating better collections of a handful of crops—crops that because of their economic importance had already been the focus of Rockefeller Foundation collections and that were arguably best represented in national and other collections—and to do so within established institutions and without global oversight. Its central objective was to ensure the long-term availability of precious resources to breeders. The FAO/IBP vision on the other hand was not limited to the core commodity crops of global agriculture but instead articulated a need for widespread conservation action, and furthermore emphasized the conservation aspects of the work over utilization. It made a strong case for the creation of new institutions, including regional seed banks and an international coordinating body. Finally, while the FAO/IBP proposal aimed at the development of an on-going effort, with permanent institutional support and activities projected for the indefinite future, foundation officers preferred to think that they were closing the door on this issue. Wortman described the World Germplasm Project as “the systematic completion of the collection of the cereal grains.”^31^

## CGIAR and the International Board for Plant Genetic Resources

Although Rockefeller Foundation administrators expressed skepticism about the ability of FAO and IBP to follow through on the ambitious scheme articulated at the FAO/IBP Technical Conference, the promoters of that scheme had not lost hope. In 1971 they laid their call for an internationally coordinated effort to conserve genetically diverse crops at the feet of a newly organized international body, the Consultative Group on International Agricultural Research (CGIAR), describing it as a concern that threatened the entire future of agricultural development. From 1971 until 1973, members of CGIAR, an organization whose mandate was to coordinate international aid for agricultural research in developing countries, grappled with the challenge of addressing this complex global conservation concern.

The origins of CGIAR, much like the rising concern about “germplasm resources,” lie in the Rockefeller Foundation’s escalating activities in the area of agricultural assistance. In the 1960s, the costs to the foundation (as well as its partner, the Ford Foundation) of operating the international agricultural research institutes—which by 1967 included CIMMYT, IRRI, the International Center for Tropical Agriculture (CIAT) and the International Institute for Tropical Agriculture (IITA)—escalated rapidly. Although the foundations wished to see these continue, they did not envision themselves as permanent financial backers. By 1969 they had initiated talks with various governments and international organizations, seeking relief from the enormous financial obligations the institutes entailed. These negotiations resulted in the 1971 formation of CGIAR, the purpose of which was to coordinate research activities that addressed the agricultural needs of developing countries. It worked primarily by directing funds from donors to the existing agricultural institutes, creating new institutes organized on the same basic model, and coordinating research efforts among all of these (CGIAR Secretariat 1971; Baum and Lejeune 1986).

From the outset, it was clear that the high-level officials who gathered as CGIAR at regular meetings would need a source of scientific and technical advice in order to effectively coordinate research at an international level. Its members therefore decided that CGIAR would have its own Technical Advisory Committee (TAC), comprising twelve scientists with expertise in agriculture and development. It was through this committee that the creation of a coordinated system to conserve crop plant diversity was presented to CGIAR as a pressing need for international agricultural research.

From the outset, the most hotly contested element of proposed genetic conservation activities was the role that would be given to FAO. The initial plan brought to the TAC in 1971 by staff of the Crop Ecology and Genetic Resources Unit of FAO called for the creation of eleven “genetic resources centres” to collect and maintain crop varieties and a coordinating body that would be “planned and supervised by internationally recognized experts,” staffed with trained personnel, and provided access to “crop specialists” to act as advisers. FAO estimated the initial five-year cost of $5 to $6.5 million to support eight or nine centers for a five-year period. It identified no new costs associated with the coordinating body—presumably because it envisioned that the existing FAO Unit would assume them. This latter assumption raised immediate objections (FAO 1971). As Otto Frankel, a central figure in the 1967 FAO/IBP Technical Conference and chair of an FAO “Panel of Experts” on plant exploration and conservation, described: “The FAO Unit concerned with genetic resources… has a staff of three professional people who are overworked and would be wholly inadequate… They have not achieved—indeed, barely attempted—the much easier task of coordinating the work of those existing institutions which could now act as such a network.”^32^

Other CGIAR members shared Frankel’s concern about FAO’s presumed leadership, especially representatives of the Rockefeller Foundation. As one foundation officer summarized, “Essentially the main points of difficulty arise from the fact that FAO would presume to bring under its control the coordinating body, and there are serious reservations about the ability of FAO to develop the strategies for a comprehensive germplasm program.” Although FAO, by virtue of its particular mandate, seemed to him a logical institution to take on international coordination, he felt that “nevertheless, FAO’s history in bureaucratic procedures and program execution leaves something to be desired.”^33^ (His assessment of course left aside the question of whether FAO had ever had the resources it needed to effectively carry out programming.) He and other administrators were quick to point out who did have such experience: the Rockefeller Foundation. Through its World Germplasm Project, the foundation had begun to coordinate efforts among international institutions—albeit those to which it was already closely connected—in order to gather and preserve crop diversity. Foundation officers felt that these activities had not been adequately taken into account in discussions about the formation of a new genetic resources network.^34^

In addition to questioning FAO’s overall capacity for program management, Rockefeller Foundation administrators raised concerns about its comparative weakness in more specialized areas of expertise. These included in particular the technical enterprises of collecting and maintaining seed stocks. To representatives of the foundation, the creation of new genetic resources centers under FAO oversight would take control out of the hands of those with the most relevant experience. In Wortman’s estimation, “Clearly, only international research centers [e.g., CIMMYT, IRRI] can really implement plans, including collection, evaluation, description, and maintenance… FAO can and would cooperate, but its officers generally would agree that action should rest with institutes, with FAO handling pub[licity]… descriptions, promotion of use of the resources, and arrangements for [international] cooperation in the evaluation of materials.”^35^

In summer 1973, after a number of TAC subcommittee meetings and several iterations of proposals, a revised plan came from FAO to CGIAR for its consideration. Surprisingly, given the tenor of the meetings and negotiations that preceded it, this proposal placed even greater control in the hands of FAO, emphasized the limitations of the existing agricultural institutes, and replaced a previously planned independent advisory panel of scientists with the FAO Panel of Experts. Unsurprisingly, Rockefeller Foundation representatives stridently objected. Once again they couched their objections in terms of their previous experience. “It seems to us that this is a scientific undertaking which must be controlled at all times… by the very best scientific judgment,” Wortman began. “Once the funds [for collecting] become available, we find literally thousands of collections coming in… One can be dealing with a bottomless pit of collection if we do not have careful scientific control.”^36^ The implication was that FAO, a political organization, would not be in a position to manage competing demands for collection from varied national and regional entities. By comparison the agricultural institutes, with their in-house scientific expertise and their prior experience in managing the “thousands of collections” rolling in, would be far better positioned.

In the wake of the 1973 proposal and ensuing discussions, a subcommittee of CGIAR finally generated terms of reference and an operational plan for an International Board for Plant Genetic Resources (IBPGR) that would be accepted by CGIAR. At the first meeting of this subcommittee, Lewis Roberts, associate director of the Rockefeller Foundation’s Agricultural Sciences program, propounded the views of the foundation. He argued first that the focus of any effort should be “the relatively few crops that supply man with most of his food” primarily on the grounds of cost, especially the cost of maintaining, evaluating, and sharing stocks. “A very sharp focus of a global genetic resources program will be required in order to keep it from becoming so broad and unwieldy that it cannot be adequately financed or managed to ensure its success.” His second major bid was for a governing board “with a high degree of autonomy,” which he thought was not ensured in the proposal of an expanded FAO unit operating in conjunction with its own Panel of Experts.^37^ According to Roberts’s account of the meeting, his proposal won favor over one advanced by FAO representatives. Roberts was subsequently asked to form a small sub-subcommittee together with members from UK and West Germany (and, notably, no one from FAO) to draft the terms of reference and plan of operation for the envisioned governing board.^38^

Although FAO had announced its intention of expanding its Crop Ecology and Genetic Resources Unit so as to be able to coordinate international conservation efforts, its central role in the finalized terms of reference was to provide a secretariat and maintain a trust fund for an independent board of experts who would be answerable primarily to CGIAR and its TAC. The activities of the IBPGR were described as giving priority to “species of major economic importance and their wild and cultivated relatives.”^39^ In this work, its chief collaborators would be the established international agricultural research institutes and several new institutions just then coming on line under the auspices of CGIAR, which would be tasked with maintaining the core world collections of key economic crops such as maize, wheat, and rice. In other words, a narrow vision that supported established approaches to and institutions for agricultural development displaced the more encompassing FAO (and earlier IBP/FAO) vision of advancing food security through the conservation of a broad range of crops and the distribution of this work and the resources necessary to support it to a range of national and international actors. This outcome reflected a broader pattern that historians have identified at FAO that began in the late 1960s and became more apparent through early 1970s, in which “the FAO lost its leadership on the issue of agricultural development” (Staples 2006, p. 121; see also Marchisio and Di Blase 1991).

The proposal hammered out by Roberts and others on the sub-committee was one that the Rockefeller Foundation was happy to support. It provided initial funds that would be used (alongside larger donations from West Germany, Sweden, Netherlands, and the United Kingdom) to get the new IBPGR up and running. In addition, Roberts—in spite of his concern in January 1974 that FAO was trying “to move in and take over major control of the Board” by orchestrating the creation of a list of candidate members of IBPGR—was invited to be among the initial twelve members, assuring the foundation a voice in all of its early proceedings.^40^

## Conclusion

Accounts by other scholars have ably demonstrated how the Rockefeller Foundation’s interventions in international agriculture were shaped by its administrators’ and personnel’s assessments of the kinds of farmers and modes of agricultural production they encountered (or imagined) in the developing world. Here I have traced instead the crucial role played by the foundation’s growing sense of its own central position in global agricultural development—and belief in its great skill in producing desirable outcomes—in shaping its activities. This, too, is an essential element to consider in understanding the history of its agricultural work in the later twentieth century.

The Rockefeller Foundation’s gradually expanding ambitions, from immediate local concerns to projected global needs, is evident in the trajectory of its involvement in seed collection and conservation. The foundation’s role in maintaining genetic stocks began as a small-scale enterprise in the 1940s, with staff in Mexico making collections of the crops they were tasked with improving for Mexican farmers. Around 1950 this initial maize collection began a slow transformation from being a working collection of Mexican maize varieties intended for use in the creation of better Mexican crops in the near future, to being the central repository for a global maize collection whose projected uses included maize breeders from around the world and especially the far future. And the Foundation did not stop at overseeing the world’s maize diversity. Where the staff of the Mexican program had begun maize collections without an initial vision for their ultimate extent, staff of newer crop improvement programs incorporated systematic collecting of regional and global diversity from the outset. In the late 1960s, longstanding concerns about the loss of genetic diversity began to be expressed with much greater urgency by many biologists and breeders. Confronting proposals for new international institutions and oversight to address this issue, Rockefeller Foundation officers presented the foundation as already well positioned to “complete” collections of genetic diversity in all the major economic crop plants and advanced the agricultural institutions they had founded as the ideal permanent international repositories for these collections. Here they had challengers, namely, FAO, which advanced itself as the organization best positioned to coordinate the international management of crop genetic resources. In the debates that followed, Rockefeller Foundation officers effectively positioned themselves and the institutions founded and funded by the foundation as better equipped than FAO, the designated UN body for agricultural concerns, to understand and take responsibility for the long-term (indeed, indefinite) maintenance of resources essential for international agricultural production.

The Rockefeller Foundation’s ever-expanding engagement with the genetic diversity of crop plants was not only a product of its officers’ growing sense of their own centrality in global agricultural production, present and future. It was also linked to a belief in the success of their own programs. Time and again they held up the work of their own employees and institutes as exemplars of collection and management of genetic material, even when they knew well that the established collections were vulnerable to disorder and decay.

The foundation’s faith in the success of its own programs can also be read into the shared belief of its administrators that they had so fundamentally transformed agricultural production that there was no hope for survival of genetically diverse varieties. This was a belief that was apparently shared among the agricultural research institutes as well, even as they became increasingly distinct from the foundation. As a document produced by a CIMMYT staff member in 1972 described, “CIMMYT, as one of the main architects of the so called ‘Green Revolution,’ has been responsible more than any other organization during the past five years for the displacement of local varieties and primitive cultivars of wheat and maize from their natural regions of cultivation and their replacement with higher yielding exotic varieties.”^41^ Rockefeller Foundation staff were not embarrassed by their institutions’ implication in this perceived destruction of genetic resources and the risks to future agricultural production thought to be attendant upon it, but rather saw it as part of their established record of success. It was simply another reason for entrusting the future of the global diversity of crop plants to the foundation and the organizations it had played a central role in creating.

## Footnotes

1 See Patel (2013) for an insightful assessment of the long history of the Green Revolution and competing claims made about its successes and failures.

2 On international efforts to conserve plant genetic resources, see Pistorius (1997) and Pistorius & Van Wijk (1999). Further studies include Saraiva (2013), Peres (2016), and Fenzi & Bonneuil (2016).

3 The earliest account of the Mexican project is Stakman et al. (1967). Subsequent scholarly accounts include Fitzgerald (1986), Jennings (1988), Cotter (2003), Matchett (2002, 2006) and Harwood (2009). For a comparison to other Mexican agricultural breeding programs of that period, see Barahona (2008).

4 Edwin Wellhausen, Oral History, Rockefeller Foundation Program in Agriculture, Volume XLII, June 1966, Rockefeller Archive Center, Rockefeller Foundation records (RF) RG 13, quotation on 29.

5 Ibid., 133.

6 For an account by one of the key collectors, see Hernández X. (1998).

7 "Annual Report, Rockefeller Agricultural Program in Mexico, February 1—December 31 Incl. 1943," RF RG 6.13, Series 1.1, Box 2, Folder 21.

8 "Review of the work of the Oficina de Estudios Especiales, S.A.G. for the year 1946–1947," RF RG 6.13, Series 1.1, Box 2, Folder 2.

9 Cleland to Miller, 17 December 1949, RF RG 1.2, Series 300, Box 1, Folder 2.

10 "Proposed Plan for the Collection and Maintenance of Native Races of Maize" (undated), Harvard University Archives, Papers of Paul C. Mangelsdorf (PCM), HUG(FP) 37.10, Box 5, Folder: National Academy of Arts and Sciences, 1954—(1 of 2).

11 Harrar to Barnard, 29 August 1951, RF RG 1.2, Series 300, Box 1, Folder 2.

12 Harrar to Barnard, 15 October 1951, RF RG 1.2, Series 300, Box 1, Folder 2.

13 Barnard to Harrar, 17 September 1951, RF RG 1.2, Series 300, Box 1, Folder 2.

14 Minutes of Meeting of Committee on Preservation of Indigenous Strains of Maize, 26 October 1951, National Academy of Sciences, Biology & Agricultural Division Files (B&A), Folder: B & A Agricultural Board Com on Preservation of Maize: Meetings, 1951–1958.

15 Committee on Preservation of Indigenous Strains of Maize, (1955), "Collections of Original Strains of Corn, II," B&A, Folder: B & A Agricultural Board Com on Preservation of Maize: Collections of Original Strains of Corn: II.

16 E.g., Harrar to Clark, 5 November 1953, RF RG 1.2, Series 300, Box 1, Folder 4; Harrar diary, 16 November 1953, RF RG 1.2, Series 300, Box 1, Folder 4.

17 Rachie to Cummings and Grant, 24 October 1957, RF RG 6.7, Series IV.5, Box 74, Folder 485.

18 Rachie to Cummings, 19 January 1960, RF RG 6.7, Series IV.5, Box 74, Folder 486.

19 A detailed account of the collecting program is K. O. Rachie, Report on the Systematic Collection of Sorghums, Millets and Maize in India, RF RG 6.7, Series IV.5, Box 81, Folder 524 (quotation on 27).

20 Gutiérrez G. to Finlay, 10 May 1976, RF RG 1.3, Series 103D, Box 18, Folder 115.

21 Most comparable collections up to this time were the product of national and imperial programs; see Flitner (2003) and Saraiva (2013) for examples.

22 Enclosure in Graves to Pino, 21 August 1973, RF RG 1.3, Series 103D, Box 16, Folder 103.

23 Wortman to Myers, 5 March 1969, RF RG 1.3, Series 103D, Box 16, Folder 101.

24 Lewis Roberts, "Request for Authorization to Proceed with the Organization of a Conference," 23 April 1971, RF RG 1.3, Series 103D, Box 17, Folder 108.

25 A good example of such referencing can be found in TAC Ad Hoc Working Group (1972).

26 See, e.g., Resolution RF 72023, 5 April 1972, RF RG 1.3, Series 103D, Box 17, Folder 106.

27 Wortman diary, 11 April 1969, RF RG 1.5, Series 200D, Box 327, Folder 2009.

28 Minutes of a Meeting on the Status of Germ Plasm Collection, Preservation, Evaluation and Utilization, 27–28 September 1971, RF RG 1.3, Series 103D, Box 16, Folder 101.

29 Ibid.

30 Resolution RF 72,023, 5 April 1972, RF RG 1.3, Series 103D, Box 17, Folder 106.

31 Wortman to Harrar, 2 March 1972, RF RG 1.3, Series 103D, Box 16, Folder 101.

32 See the additional pages appended to FAO (1971) entitled "Comments on Dr. León's statement for TAC."

33 Pino, memo, 15 December 1972, RF, RG 1.3, Subseries 103D, Box 16, Folder 101.

34 Roberts, memo to Pino, 27 March 1972, RF, RG 1.3, Subseries 103D, Box 16, Folder 101.

35 Wortman, memo, 31 January 1972, RF, RG 1.3, Subseries 103D, Box 16, Folder 101.

36 Enclosure in Graves to Pino, 21 August 1973, RAC, RF, RG 1.3, Subseries 103D, Box 16, Folder 103.

37 Roberts, "The Collection, Preservation and Evaluation of the World's Plant Genetic Resources," 17 September 1973, RF RG 1.3, Subseries 103D, Box 16, Folder 103.

38 Roberts, memo [on meeting of Sub-Committee on Genetic Resources], n.d., RF, RG 1.3, Subseries 103D, Box 16, Folder 104.

39 IBPGR, "Terms of Reference and Operational Rules and Procedures of the International Board for Plant Genetic Resources," 1974, RF, RG 1.3, Subseries 103D, Box 20, Folder 127.

40 Roberts, 7 January 1974, RF RG 12, Lewis Roberts Officer Diary, Box 413, File 1974.

41 Enclosure in Finlay to Roberts, 17 March 1972, RF RG 1.3, Series 103D, Box 18, Folder 112.

## References

[CR1] Barahona, A. (2008). Mendelism and agriculture in the first decades of the XXth Century in Mexico. In *A cultural history of heredity IV: Heredity in the century of the gene*, Preprint 343 (pp. 111–128). Berlin: Max-Planck-Institut für Wissenschaftsgeschichte.

[CR2] Baranski, M. (2015). *The wide adaptation of green revolution wheat*. Ph.D. thesis, Arizona State University. 10.1016/j.shpsc.2015.01.004 25655670

[CR3] Baum WG, Lejeune ML (1986). Partners against hunger: The Consultative Group on International Agricultural Research.

[CR4] CGIAR Secretariat. (1971). *Resolution: Objectives, composition and organizational structure*. May. http://hdl.handle.net/10947/5254.

[CR6] Chandler, R. F. Jr. (1992). *An adventure in applied science: A history of the International Rice Research Institute*. Manila: IRRI. http://books.irri.org/9711040638_content.pdf.

[CR7] Clark JA (1956). Collection, preservation, and utilization of indigenous strains of maize. Economic Botany.

[CR8] Cotter JE (2003). Troubled harvest: Agronomy and revolution in Mexico, 1880–2002.

[CR9] Cullather N (2004). Miracles of modernization: The green revolution and the apotheosis of technology. Diplomatic History.

[CR10] Cullather N (2010). The hungry world: America’s cold war battle against poverty in Asia.

[CR11] Curry, H. A. (2017). Breeding uniformity and banking diversity: The genescapes of industrial agriculture, 1935–1970. *Global Environment, 10*, 83–113.

[CR12] FAO. (1971). *Proposal to establish a network of genetic resources centres*. CGIAR TAC, Second Meeting, Rome, October 19–22. http://hdl.handle.net/10947/973.

[CR14] Fenzi M, Bonneuil C (2016). From “genetic resources” to “ecosystem services”: A century of science and global policies for crop diversity conservation. Culture, Agriculture, Food and Environment.

[CR15] Fitzgerald D (1986). Exporting American agriculture: The Rockefeller Foundation in Mexico, 1943–1953. Social Studies of Science.

[CR16] Flitner M (2003). Genetic geographies: A historical comparison of agrarian modernization and eugenic thought in Germany, the soviet union, and the United States. Geoforum.

[CR17] Frankel OH, Bennett E (1970). Genetic resources in plants—Their exploration and conservation. IBP Handbook No. 11.

[CR18] Frankel OH, Bennett E, Frankel OH, Bennett E (1970). Genetic resources. Genetic resources in plants—Their exploration and conservation, IBP Handbook No. 11.

[CR19] Harwood Jonathan (2009). Peasant friendly plant breeding and the early years of the green revolution in Mexico. Agricultural History.

[CR20] Harwood Jonathan (2012). Europe’s green revolution and others since: The rise and fall of peasant-friendly plant breeding.

[CR21] Hernández X., E. (1998). Experiences in the collection of maize germplasm. In *CIMMYT*, *Recent advances in the conservation and utilization of genetic resources: Proceedings of the global maize germplasm workshop* (pp. 1–8). Mexico, DF: CIMMYT.

[CR22] House L (1980). A guide to sorghum breeding.

[CR23] Jennings B (1988). Foundations of international agricultural research: Science and politics in Mexican agriculture.

[CR24] Mangelsdorf PC (1974). Corn: Its origin, evolution, and improvement.

[CR25] Marchisio, S., & Di Blase, A. (1991). *The Food and Agriculture Organization (FAO)*. Dordrecht: Martinus Nijhoff Publishers.

[CR26] Matchett, K. (2002). *Plant sciences research and agriculture in Mexico: Tensions and collaboration among Mexican and U.S. scientists, 1935–1965*. Ph.D. thesis, University of Minnesota.

[CR27] Matchett K (2006). At odds over inbreeding: An abandoned attempt at Mexico/United States collaboration to “improve” Mexican corn, 1940–1950. Journal of the History of Biology.

[CR28] Nally D, Taylor S (2015). The politics of self-help: The Rockefeller Foundation, philanthropy and the “long” green revolution. Political Geography.

[CR29] Oasa, E. K. (1981). *The international rice research institute and the green revolution: A case study on the politics of agricultural research*. Ph.D. Thesis, University of Hawaii.

[CR30] Patel R (2013). The long green revolution. Journal of Peasant Studies.

[CR31] Peres S (2016). Saving the gene pool for the future: Seed banks as archives. Studies in History and Philosophy of Science-Part C.

[CR32] Perkins JH (1997). Geopolitics and the green revolution: Wheat, genes, and the Cold War.

[CR33] Pistorius R (1997). Scientists, plants, and politics: A history of the plant genetic resources movement.

[CR34] Pistorius R, Van Wijk J (1999). The exploitation of plant genetic information: Political strategies in crop development.

[CR35] Saraiva T, Disco N, Kranakis E (2013). Breeding Europe: Crop diversity, gene banks, and commoners. Cosmopolitan commons: Sharing resources and risks across borders.

[CR36] Shepherd CJ (2005). Imperial science: The Rockefeller Foundation and agricultural science in Peru, 1940–1960. Science as Culture.

[CR37] Smith E (2009). Imaginaries of development: The Rockefeller Foundation and rice research. Science as Culture.

[CR38] Stakman EC, Bradfield R, Mangelsdorf PC (1967). Campaigns against hunger.

[CR39] Staples A (2006). The birth of development: How the world bank, Food and Agriculture Organization, and World Health Organization changed the world, 1945–1965.

[CR40] Taba S, van Ginkel M, Hoisington D, Poland D (2004). Wellhausen–Anderson plant genetic resources center: Operations manual.

[CR41] TAC Ad Hoc Working Group. (1972). *The collection, evaluation and conservation of plant genetic resources*. CGIAR TAC Third Meeting, Rome, 10–13 April. http://hdl.handle.net/10947/1523.

[CR42] Wellhausen EJ, Roberts LM, Hernandez XE, Mangelsdorf PC (1952). Races of maize in Mexico: Their origin characteristics and distribution.

